# Supplementary material for: Resilience during the COVID-19 pandemic: Associations with changes in burnout and mental well-being among NHS mental health staff in England
Source: PLoS One. 2025 Jul 8;20(7):e0326753. doi: 10.1371/journal.pone.0326753 (PMC12237035; doi:10.1371/journal.pone.0326753)
Supplement: S1 Table — (DOCX) [file pone.0326753.s001.docx]

**S1 Table.** **Mixed Model Coefficients.**

|  | Burnout | | | Mental well-being | | |
| --- | --- | --- | --- | --- | --- | --- |
| VARIABLES | Initial outbreak | Initial easing | Second outbreak | Initial outbreak | Initial easing | Second outbreak |
| Time | -0.13 | -0.09 | 0.03 | 14.02** | -7.66** | 3.24 |
|  | (0.11) | (0.16) | (0.07) | (3.96) | (2.62) | (4.95) |
| Resilience at baseline | -0.07** | -0.06** | -0.05** | 0.72** | 0.77** | 0.79** |
|  | (0.01) | (0.01) | (0.01) | (0.15) | (0.09) | (0.07) |
| Time X Resilience | 0.01* | 0.01 | 0.01** | -0.06 | -0.08 | -0.07 |
| interaction | (0.00) | (0.01) | (0.00) | (0.16) | (0.05) | (0.04) |
| Constant | 3.32** | 3.33** | 2.94** | 6.86 | 29.55** | 7.43 |
|  | (0.16) | (0.19) | (0.19) | (4.89) | (7.05) | (4.98) |

Robust standard errors in parentheses

** p<0.01, * p<0.05
