# Supplementary material for: Resilience during the COVID-19 pandemic: Associations with changes in burnout and mental well-being among NHS mental health staff in England
Source: PLoS One. 2025 Jul 8;20(7):e0326753. doi: 10.1371/journal.pone.0326753 (PMC12237035; doi:10.1371/journal.pone.0326753)
Supplement: S2 Appendix — (DOCX) [file pone.0326753.s002.docx]

**S2 Appendix. Details regarding data-driven process to select covariates.**

*Burnout analyses*

*Group: initial peak*

Base model fit: BIC = -81.60, AIC = -115.40

|  | Significant main effect | Significant interaction with time | Model fit indices | Final decision |
| --- | --- | --- | --- | --- |
| Sex | No | No | n/a | Don’t include |
| Age | Yes | Yes | BIC = -88.12, AIC = -185.99 | Poor model fit – don’t include |
| Ethnicity | Yes | Yes | BIC = -27.58, AIC = -124.18 | Better model fit - include |
| Relationship status | Yes | No | BIC = -68.20, AIC = -150.31 | Better model fit - include |
| Time since professional registration | Yes | Yes | BIC = -47.53, AIC = -187.53 | Better model fit - include |
| Family support | Yes | Yes | BIC = -148.69, AIC = -245.17 | Poor model fit – don’t include |
| Manager support | Yes | Yes | BIC = -221.31, AIC = -317.86 | Poor model fit – don’t include |
| Colleague support | Yes | Yes | BIC = -247.30, AIC = -150.7 | Poor model fit – don’t include |
| COVID symptoms | No | Yes | BIC = -69.37, AIC = -136.77 | Better model fit - include |

*Group: Initial easing*

Base model fit: BIC = -6008.10, AIC = -6046.07

|  | Significant main effect | Significant interaction with time | Model fit indices | Final decision |
| --- | --- | --- | --- | --- |
| Sex | Yes | Yes | BIC = -5996.68, AIC = -6078.05 | Poor model fit – don’t include |
| Age | No | Yes | BIC = -6306.86, AIC = -6414.54 | Poor model fit – don’t include |
| Ethnicity | Yes | Yes | BIC = -6118.96, AIC = -6227.40 | Poor model fit – include for consistency |
| Relationship status | Yes | No | BIC = -6041.40, AIC = -6133.55 | Poor model fit – include for consistency |
| Time since professional registration | Yes | Yes | BIC = -6118.96, AIC = -6227.40 | Poor model fit – include for consistency |
| Family support | Yes | Yes | BIC = -6466.57, AIC = -6574.96 | Poor model fit – don’t include |
| Manager support | Yes | Yes | BIC = -6450.06, AIC = -6558.41 | Poor model fit – don’t include |
| Colleague support | Yes | Yes | BIC = -6229.90, AIC = -6338.25 | Poor model fit – don’t include |
| COVID symptoms | No | No | BIC = -5973.50, AIC = -6049.26 | Better model fit - include |

*Group: Second peak*

Base model fit: BIC = -11622.39, AIC = -11661.86

|  | Significant main effect | Significant interaction with time | Model fit indices | Final decision |
| --- | --- | --- | --- | --- |
| Sex | Yes | Yes | BIC = -11823.12, AIC = -11907.68 | Poor model fit – don’t include |
| Age | No | Yes | BIC = -11771.52, AIC = -11883.57 | Poor model fit – don’t include |
| Ethnicity | Yes | Yes | BIC = -11701.37, AIC = -11797.15 | Poor model fit – include for consistency |
| Relationship status | Yes | No | BIC = -11882.60, AIC = -11978.35 | Poor model fit – include for consistency |
| Time since professional registration | Yes | Yes | BIC = -14202.04, AIC = -14365.49 | Poor model fit – include for consistency |
| Family support | No | No | n/a | Don’t include |
| Manager support | Yes | Yes | BIC = -13230.78, AIC = -13118.02 | Poor model fit – don’t include |
| Colleague support | Yes | No | BIC = -11978.73, AIC = -12085.85 | Poor model fit – don’t include |
| COVID symptoms | No | No | BIC = -12169.24, AIC = -12247.98 | Poor model fit – include for consistency |

*Well-being analyses*

*Group: initial peak*

Base model fit: BIC = 15550.59, AIC = 15516.66

|  | Significant main effect | Significant interaction with time | Model fit indices | Final decision |
| --- | --- | --- | --- | --- |
| Sex | No | No | n/a | Don’t include |
| Age | No | Yes | BIC = 15338.31, AIC = 15241.85 | Better model fit - include |
| Ethnicity | Not converged | Not converged | BIC = 15576.85, AIC = 15479.91 | Better model fit - include |
| Relationship status | Yes | No | BIC = 15585.94, AIC = 15503.54 | Better model fit - include |
| Time since professional registration | Yes | Yes | BIC = 15513.91, AIC = 15373.41 | Better model fit - include |
| Family support | Yes | Yes | BIC = 15445.39, AIC = 15348.53 | Better model fit - include |
| Manager support | Yes | Yes | BIC = 15521.18, AIC = 15424.28 | Better model fit - include |
| Colleague support | Yes | Yes | BIC = 15488.54, AIC = 15391.60 | Better model fit - include |
| COVID symptoms | Yes | No | BIC = 15058.04, AIC = 14990.41 | Better model fit - include |

*Group: initial easing*

Base model fit: BIC = 38772.38, AIC = 38734.31

|  | Significant main effect | Significant interaction with time | Model fit indices | Final decision |
| --- | --- | --- | --- | --- |
| Sex | No | Yes | BIC = 38474.24, AIC = 38392.65 | Slightly improved model fit – exclude for consistency |
| Age | No | Yes | BIC = 36479.55, AIC = 36371.55 | Better model fit - include |
| Ethnicity | Yes | Yes | BIC = 38026.88, AIC = 37918.13 | Better model fit - include |
| Relationship status | Yes | Yes | BIC = 38012.49, AIC = 37920.07 | Better model fit - include |
| Time since professional registration | No | Yes | BIC = 38330.18, AIC = 38172.52 | Better model fit - include |
| Family support | Yes | Yes | BIC = 37949.92, AIC = 37841.24 | Better model fit - include |
| Manager support | Yes | Yes | BIC = 37480.96, AIC = 37372.31 | Better model fit - include |
| Colleague support | Yes | Yes | BIC = 37971.82, AIC = 37863.18 | Better model fit - include |
| COVID symptoms | No | No | BIC = 38077.87, AIC = 38001.90 | Better model fit - include |

*Group: Second peak*

Base model fit: BIC = 32890.94, AIC = 32851.35

|  | Significant main effect | Significant interaction with time | Model fit indices | Final decision |
| --- | --- | --- | --- | --- |
| Sex | Yes | Yes | BIC = 32784.35, AIC = 32699.52 | Slightly improved model fit – exclude for consistency |
| Age | Yes | Yes | BIC = 31944.49, AIC = 31832.09 | Better model fit - include |
| Ethnicity | Yes | Yes | BIC = 32785.62, AIC = 32678.24 | Better model fit - include |
| Relationship status | Yes | No | BIC = 32639.47, AIC = 32543.40 | Better model fit - include |
| Time since professional registration | No | Yes | BIC = 31082.18, AIC = 30918.20 | Better model fit - include |
| Family support | Yes | No | BIC = 32404.26, AIC = 32296.84 | Better model fit - include |
| Manager support | Yes | No | BIC = 32429.87, AIC = 32316.74 | Better model fit - include |
| Colleague support | Yes | Yes | BIC = 32453.05, AIC = 32345.58 | Better model fit - include |
| COVID symptoms | No | No | BIC = 32220.95, AIC = 32141.97 | Better model fit - include |
