# Supplementary material for: Resilience during the COVID-19 pandemic: Associations with changes in burnout and mental well-being among NHS mental health staff in England
Source: PLoS One. 2025 Jul 8;20(7):e0326753. doi: 10.1371/journal.pone.0326753 (PMC12237035; doi:10.1371/journal.pone.0326753)
Supplement: Attachment — Submitted filename: PONE-D-24-44533_reviewer_(due_25_Jan).pdf [file pone.0326753.s003.pdf]

PLOS ONE

Resilience during the COVID-19 pandemic: Associations with changes in burnout and mental well-being among NHS mental health staff in England

--Manuscript Draft--

**Manuscript Number:**	PONE-D-24-44533
**Article Type:**	Research Article
**Full Title:**	Resilience during the COVID-19 pandemic: Associations with changes in burnout and mental well-being among NHS mental health staff in England
**Short Title:**	Resilience in mental health staff during COVID-19
**Corresponding Author:**	Natalia Kika<br>King's College London Institute of Psychiatry Psychology & Neuroscience<br>London, UNITED KINGDOM OF GREAT BRITAIN AND NORTHERN IRELAND
**Keywords:**	Occupational Psychiatry; mental health; Health Service Research; Psychiatric Services; resilience; burnout
**Abstract:**	Background<br><br>The pressures of the COVID-19 pandemic placed mental healthcare professionals at an increased risk of burnout and decreased mental wellbeing. Resilience strategies have been put in place as a protective measure, but little is known about how mental wellbeing evolved over time in this group and how resilience affects it. This study aimed to: 1) investigate long-term changes in burnout and mental well-being among mental healthcare professionals working in the National Health Service (NHS) during the COVID-19 pandemic in the United Kingdom (UK); and 2) examine whether baseline resilience levels predicted decreased burnout and increased mental well-being over the course of the pandemic.<br><br>Methods<br><br>The study used data from NHS CHECK, a longitudinal cohort study investigating NHS staff mental health and well-being since the COVID-19 pandemic. Clinical mental health staff (n = 3,289) who completed self-report measures at three time points (baseline, 6 and 12 months later). Baseline surveys were conducted during the initial pandemic peak (April 2020 – June 2020; n = 543), the initial easing of restrictions (July 2020 – September 2020; n = 1,098), and the second peak (October 2020 – January 2021, n = 1,648).<br><br>Results<br><br>Mixed model analyses showed that burnout scores increased over time, with higher resilience at baseline predicting lower burnout 6 and 12 months later. However, rises in burnout were most pronounced in the high resilience group. Well-being remained relatively stable over time, with staff with higher resilience at baseline reporting higher well-being over time.<br><br>Conclusions<br><br>Resilience was linked with both lower burnout and higher well-being in NHS mental health staff throughout the COVID-19 pandemic. However, staff with initially high levels of resilience reported greater increases in burnout symptoms compared to those with lower levels of resilience scores.
**Order of Authors:**	Natalia Kika
	Nora Trompeter
	Danielle Lamb
	Rupa Bhundia
	Ewan Carr

Powered by Editorial Manager® and ProduXion Manager® from Aries Systems Corporation

	Brendan Dempsey
	Neil Greenberg
	Ira Madan
	Christopher Penfold
	Rosalind Raine
	Simon Wessely
	Sharon A.M. Stevelink
**Additional Information:**	
**Question**	**Response**
**Financial Disclosure**<br><br>Enter a financial disclosure statement that describes the sources of funding for the work included in this submission. Review the submission guidelines for detailed requirements. View published research articles from *PLOS ONE* for specific examples.<br><br>This statement is required for submission and **will appear in the published article** if the submission is accepted. Please make sure it is accurate.<br><br>**Funded studies**<br>Enter a statement with the following details:<br>• Initials of the authors who received each award<br>• Grant numbers awarded to each author<br>• The full name of each funder<br>• URL of each funder website<br>• Did the sponsors or funders play any role in the study design, data collection and analysis, decision to publish, or preparation of the manuscript?<br><br>Did you receive funding for this work?	Yes
Please add funding details.<br>as follow-up to "**Financial Disclosure**<br><br>Enter a financial disclosure statement that describes the sources of funding for the work included in this submission. Review the submission guidelines for detailed requirements. View published research articles from *PLOS ONE* for specific	Funding for NHS CHECK was received from the following sources: Medical Research Council (MR/V034405/1); UCL/Wellcome (ISSF3/ H17RCO/C3); Rosetrees Trust (M952 and PGL22/100103); Economic and Social Research Council (ES/V009931/1); NIHR ARC North Thames; NHS England and NHS Improvement; Manolo Blahnik International; Koa Health; Colt Foundation; as well as seed funding from National Institute for Health Research Maudsley Biomedical Research Centre, King's College London, National Institute for Health and Care Research Health Protection Research Unit in Emergency Preparedness and Response at King's College London.

examples.<br><br>This statement is required for submission and **will appear in the published article** if the submission is accepted. Please make sure it is accurate.<br><br>**Funded studies**<br>Enter a statement with the following details:<br>• Initials of the authors who received each award<br>• Grant numbers awarded to each author<br>• The full name of each funder<br>• URL of each funder website<br>• Did the sponsors or funders play any role in the study design, data collection and analysis, decision to publish, or preparation of the manuscript?<br><br>Did you receive funding for this work?"	
Please select the country of your main research funder (please select carefully as in some cases this is used in fee calculation).<br>as follow-up to "**Financial Disclosure**<br><br>Enter a financial disclosure statement that describes the sources of funding for the work included in this submission. Review the submission guidelines for detailed requirements. View published research articles from *PLOS ONE* for specific examples.<br><br>This statement is required for submission and **will appear in the published article** if the submission is accepted. Please make sure it is accurate.	UNITED KINGDOM - GB

Powered by Editorial Manager® and ProduXion Manager® from Aries Systems Corporation

**Funded studies**<br>Enter a statement with the following details:<br>• Initials of the authors who received each award<br>• Grant numbers awarded to each author<br>• The full name of each funder<br>• URL of each funder website<br>• Did the sponsors or funders play any role in the study design, data collection and analysis, decision to publish, or preparation of the manuscript?<br><br>Did you receive funding for this work?"	
**Competing Interests**<br><br>Use the instructions below to enter a competing interest statement for this submission. On behalf of all authors, disclose any competing interests that could be perceived to bias this work—acknowledging all financial support and any other relevant financial or non-financial competing interests.<br><br>This statement is required for submission and **will appear in the published article** if the submission is accepted. Please make sure it is accurate and that any funding sources listed in your Funding Information later in the submission form are also declared in your Financial Disclosure statement.<br><br>View published research articles from *PLOS ONE* for specific examples.	The authors have declared that no competing interests exist.

Powered by Editorial Manager® and ProduXion Manager® from Aries Systems Corporation

**NO authors have competing interests**<br><br>Enter: *The authors have declared that no competing interests exist.*<br><br>**Authors with competing interests**<br><br>Enter competing interest details beginning with this statement:<br><br>*I have read the journal's policy and the authors of this manuscript have the following competing interests: [insert competing interests here]*<br><br>* typeset	
**Ethics Statement**<br><br>Enter an ethics statement for this submission. This statement is required if the study involved:<br><br>• Human participants<br>• Human specimens or tissue<br>• Vertebrate animals or cephalopods<br>• Vertebrate embryos or tissues<br>• Field research<br><br>Write "N/A" if the submission does not require an ethics statement.<br><br>General guidance is provided below. Consult the submission guidelines for detailed instructions. **Make sure that all information entered here is included in the Methods section of the manuscript.**	Ethical approval for NHS CHECK was granted by the Health Research Authority (reference: 20/HRA/2107, IRAS: 282686) and local Trust Research and Development approval. Written consent was obtained at each study wave.

Powered by Editorial Manager® and ProduXion Manager® from Aries Systems Corporation

**Format for specific study types**<br><br>**Human Subject Research (involving human participants and/or tissue)**<br>• Give the name of the institutional review board or ethics committee that approved the study<br>• Include the approval number and/or a statement indicating approval of this research<br>• Indicate the form of consent obtained (written/oral) or the reason that consent was not obtained (e.g. the data were analyzed anonymously)<br><br>**Animal Research (involving vertebrate animals, embryos or tissues)**<br>• Provide the name of the Institutional Animal Care and Use Committee (IACUC) or other relevant ethics board that reviewed the study protocol, and indicate whether they approved this research or granted a formal waiver of ethical approval<br>• Include an approval number if one was obtained<br>• If the study involved *non-human primates*, add *additional details* about animal welfare and steps taken to ameliorate suffering<br>• If anesthesia, euthanasia, or any kind of animal sacrifice is part of the study, include briefly which substances and/or methods were applied<br><br>**Field Research**<br><br>Include the following details if this study involves the collection of plant, animal, or other materials from a natural setting:<br>• Field permit number<br>• Name of the institution or relevant body that granted permission	
**Data Availability**<br><br>Authors are required to make all data underlying the findings described fully available, without restriction, and from the time of publication. PLOS allows rare exceptions to address legal and ethical concerns. See the PLOS Data Policy and FAQ for detailed information.	No - some restrictions will apply

A Data Availability Statement describing where the data can be found is required at submission. Your answers to this question constitute the Data Availability Statement and **will be published in the article**, if accepted.<br><br>**Important:** Stating 'data available on request from the author' is not sufficient. If your data are only available upon request, select 'No' for the first question and explain your exceptional situation in the text box.<br><br>Do the authors confirm that all data underlying the findings described in their manuscript are fully available without restriction?	
**Describe where the data may be found in full sentences. If you are copying our sample text, replace any instances of XXX with the appropriate details.**<br><br>• If the data are **held or will be held in a public repository**, include URLs, accession numbers or DOIs. If this information will only be available after acceptance, indicate this by ticking the box below. For example: *All XXX files are available from the XXX database (accession number(s) XXX, XXX.*).<br>• If the data are all contained **within the manuscript and/or Supporting Information files**, enter the following: *All relevant data are within the manuscript and its Supporting Information files.*<br>• If neither of these applies but you are able to provide **details of access elsewhere**, with or without limitations, please do so. For example:<br><br>*Data cannot be shared publicly because of [XXX]. Data are available from the XXX Institutional Data Access / Ethics Committee (contact via XXX) for researchers who meet the criteria for access to confidential data.*<br><br>*The data underlying the results presented in the study are available from (include the name of the third party*	Due to Research Ethics Committee restrictions, the data set is not publicly available. Requests to access to the de-identified data set can be made to the NHS CHECK team at nhscheck@kcl.ac.uk.Data will be available to researchers who provide a justified hypothesis and structured statistical analysis plan addressing a legitimate research question that is approved by the NHS CHECK Senior Research Team and after the signing of a data sharing agreement. Only deidentified participant data will be provided.

and contact information or URL).<br>• This text is appropriate if the data are owned by a third party and authors do not have permission to share the data.<br><br>* typeset	
Additional data availability information:	

Powered by Editorial Manager® and ProduXion Manager® from Aries Systems Corporation

Manuscript

Click here to access/download;Manuscript;NHS CHECK Resilience Paper Final.docx

Resilience during the COVID-19 pandemic: Associations with changes in burnout and mental well-being among NHS mental health staff in England

Short title: Resilience in mental health staff during COVID-19

Natalia Kika[1* ¶], Nora Trompeter[1¶], Danielle Lamb[2], Rupa Bhundia[1], Ewan Carr[3], Brendan Dempsey[2], Neil Greenberg[1], Ira Madan[4], Christopher Penfold[5], Rosalind Raine[2], Simon Wessely[1], Sharon A.M. Stevelink[1]

[1]King's Centre for Military Health Research, Institute of Psychiatry, Psychology & Neuroscience, King's College London, United Kingdom, 10 Cutcombe Road, London SE5 9RJ, UK

[2] Department of Applied Health Research, University College London, London, UK

[3] Department of Biostatistics & Health Informatics, Institute of Psychiatry, Psychology & Neuroscience, King's College London, London

[4] Guy's and St Thomas' NHS Trust, London, UK

[5] National Institute for Health Research Applied Research Collaboration West (NIHR ARC West), University Hospitals Bristol and Weston NHS Foundation Trust, Bristol, UK

*Corresponding author

Email: Natalia.kika@kcl.ac.uk

[¶]These authors contributed equally to this work.

Abstract

Background

The pressures of the COVID-19 pandemic placed mental healthcare professionals at an increased risk of burnout and decreased mental wellbeing. Resilience strategies have been put in place as a protective measure, but little is known about how mental wellbeing evolved over time in this group and how resilience affects it. This study aimed to: 1) investigate long-term changes in burnout and mental well-being among mental healthcare professionals working in the National Health Service (NHS) during the COVID-19 pandemic in the United Kingdom (UK); and 2) examine whether baseline resilience levels predicted decreased burnout and increased mental well-being over the course of the pandemic.

Methods

The study used data from NHS CHECK, a longitudinal cohort study investigating NHS staff mental health and well-being since the COVID-19 pandemic. Clinical mental health staff (*n* = 3,289) who completed self-report measures at three time points (baseline, 6 and 12 months later). Baseline surveys were conducted during the initial pandemic peak (April 2020 – June 2020; *n* = 543), the initial easing of restrictions (July 2020 – September 2020; *n* = 1,098), and the second peak (October 2020 – January 2021, *n* = 1,648).

Results

Mixed model analyses showed that burnout scores increased over time, with higher resilience at baseline predicting lower burnout 6 and 12 months later. However, rises in burnout were most pronounced in the high resilience group. Well-being remained relatively stable over time, with staff with higher resilience at baseline reporting higher well-being over time.

Conclusions

Resilience was linked with both lower burnout and higher well-being in NHS mental health staff throughout the COVID-19 pandemic. However, staff with initially high levels of resilience reported greater increases in burnout symptoms compared to those with lower levels of resilience scores.

Introduction

The COVID-19 pandemic has posed severe challenges for mental health staff due to staff shortages, increased demand in services, and challenges of remote working [1]. Implementation of infection-control measures in mental health settings resulted not only in changed working conditions but also in reduced patient care [2]. These included cancelled outpatient appointments, reduced group sizes in group therapy, decreased inpatient services, and some hospitals turning psychiatric wards into COVID-wards [3-6]. Thus, the practical limitations in the delivery of mental health care, while the need for mental health services continued to increase due to the additional burden of the pandemic [7], placed mental health staff under increased pressure and at risk of burnout and decreased mental well-being [1]. Critically, some of the issues remain despite the acute stages of the pandemic easing, with an increasing workload and a backlog of referrals for mental health staff as the pandemic has progressed [8] It is therefore crucial to consider factors that can both reduce the risk of burnout and increase mental well-being among mental health staff. This study will examine the longer-term changes in burnout and mental well-being of mental health staff during the pandemic and consider whether resilience, defined as the ability to bounce back from setbacks during challenging times [9], decreased burnout and increased mental well-being.

Burnout has long been regarded as a critical issue among mental health staff [10] and while it is not classified as a medical condition, it is defined as an occupational syndrome in the International Classification of Diseases (ICD-11) [11]. With the onset of the COVID-19 pandemic there has been an increased focus on burnout and mental well-being among caring professionals due to the pandemic pressures adding to existing high workloads [12]. Further, there are concerns that changes experienced during the pandemic will have long-lasting impacts. Qualitative research among mental health nurses showed that while many concerns raised by staff related to the immediate impact of COVID-specific concerns (e.g., risk of infection), mental health nurses also reported several concerns relating to more long-term issues brought on by the pandemic (e.g., increased mental health needs among patients) [13]. Similarly, another study found that a fear of

infection and a lack of organisational support were determinants of burnout among mental health staff during the pandemic [14]. As such, it is important to consider both the prevention of burnout and the promotion of mental well-being among mental health staff. One common factor examined in the literature regarding this aspect has been psychological resilience [15].

Resilience refers to the ability of individuals to positively adapt to adverse events or experiences and is considered an acquired skill as opposed to a stable trait [16], making it a valuable intervention target. Data collected during the early stages of the pandemic suggest that higher resilience levels were linked to lower depression, stress and anxiety in healthcare staff [17]. Similarly, resilience has been linked to lower intention to leave work among healthcare staff [18], lower anxiety [19], lower likelihood of meeting criteria for an anxiety disorder or depression [20], as well as higher quality of life [21]. This cross-sectional research suggests that resilience may be related to lower burnout and higher mental well-being among healthcare staff. However, given that resilience is, in part, characterised by an ability to "bounce back" and adapt to changing circumstances, some initial distress in response to a stressor is expected [22]. Thus, longitudinal research is required to examine whether resilience is indeed linked to a longer-term adaptation during stressful experiences (e.g., during the pandemic). Initial research on mental distress during different stages of the pandemic seems to suggest that healthcare workers may have indeed "bounced back" from the negative effects of the pandemic [23]. While healthcare workers in Spain reported higher levels of anxiety, depression and stress compared to the general public in March 2020, they reported lower levels of anxiety, depression and stress compared to the general public in March 2021 [24]. In the UK, cross-sectional research has shown the proportion of healthcare workers reporting high levels of depression and anxiety symptoms at a four-fold increase compared to pre-pandemic levels with resilience among the factors associated with lower psychiatric symptoms [25]. However, as a systematic review examining resilience, social support, and coping behaviours among healthcare workers in the first year of the pandemic concluded, moderate to high resilience levels reported in the first year of the pandemic may have evolved differently throughout

the pandemic, and as such, longitudinal evidence is required to examine such patterns in mental healthcare staff specifically [26].

Emerging longitudinal research supports the existence of varied patterns. Thus, some research demonstrates that mental distress increased over the course of the pandemic among individuals with low or normal levels of resilience, but no change was observed among individuals with high levels of resilience [27]. In contrast, research among healthcare workers in the United States found no significant relationship between resilience at the start of the pandemic (March 2020) and changes in psychological distress after three months [23]. Thus, there is currently no clear evidence that resilience is linked with either decreased burnout or increased mental well-being, and no existing research has examined this prospective relationship among mental health staff whose challenges may be specific to their roles.

Despite the lack of evidence, many organisations quickly adopted resilience interventions during the pandemic to increase staff well-being [28-29]. While some of these interventions have shown promising effects [30], a systematic review concluded that there is currently very low-certainty evidence about the effectiveness of such interventions [31]. Additionally, placing emphasis on personal characteristics (e.g., resilience), rather than structural characteristics (e.g., organisational support and work environment), might be counter-productive in reducing burnout and increasing mental well-being, with research showing that organisational, rather than individual factors, determine burnout in mental health staff during the pandemic [14]. This criticism is not new or unique to the pandemic. As previous scholars have observed, resilience interventions may inadvertently place responsibility back on individuals instead of tackling bigger organisational or societal challenges [32]. Further, implementing interventions which aim to impact factors that have not yet been shown to decrease burnout or increase mental well-being is pre-emptive at best.

Encouraging the use of reportedly resilience-enhancing interventions may also place additional pressure on mental health staff with limited support systems in place for their own well-

being. A study evaluating a resilience coaching programme during the COVID-19 pandemic in Canada found that mental health staff were a crucial source of support to their colleagues working in acute care settings [33]. Simultaneously, a qualitative study interviewing mental healthcare workers in the UK who supported their colleagues working in front-line settings found that, although it was rewarding to support front-line colleagues during the peak of the pandemic, there was not enough training or knowledge on how to support them during this time [34]. Mental health staff also reported unsustainable working patterns to deliver support to their acute care colleagues, and a lack of support for themselves, especially having been exposed to accounts of potentially traumatic or morally injurious events from their colleagues, defined as singular events which violate one's moral or ethical code [35].

The current study sought to address these critical gaps in the literature by examining long-term changes in burnout and mental well-being among mental health staff working within the National Health Service (NHS) during the pandemic. Importantly, we examined outcomes for participants recruited during three critical time periods of the pandemic in the UK: The initial outbreak (April 2020 – June 2020), initial easing of restrictions (July 2020 – September 2020), second outbreak (October 2020 – January 2021). For those recruited during each period, we considered whether baseline resilience was linked with subsequent decreases in burnout and increases in mental well-being after 6 and 12 months.

Methods

Participants and procedure

We used data from the NHS CHECK study [36], a longitudinal cohort study of the mental health and well-being of health care staff during the COVID-19 pandemic in England. In brief, the study recruited NHS staff from 18 participating Trusts to participate in a longitudinal study on the effects of the pandemic. All staff (clinical and non-clinical) were invited to complete an online survey between 27/04/2020 and 15/01/2021. Participants were then re-invited to complete surveys 6

(27/10/2020 until 15/08/2021) and 12 months (27/04/2021 until 21/02/2022) after their initial response, with a four-week window to complete it. As part of the survey all participants had the option to either complete a short or long version to minimise participant burden. Details on the methodology are available in the NHS CHECK protocol [36].

To address the evidence gaps outlined above, we included all staff who were identified as clinical mental health staff. That is, psychologists, assistant psychologists, psychiatrists, psychiatric nurses, or other clinical staff working in psychological services, psychiatry, or secure settings (n = 6,322). As the current study used measures that were only included in the long version of the questionnaire, we restricted analyses to participants who completed the long survey at baseline (n = 3,289). Mental health staff who completed the long survey at baseline were more likely to be female ($p < .001$, $V = .05$), White ($p < .001$, $V = .11$), and older ($p = .01$, $V = .05$). However, effect sizes were weak indicating few meaningful differences between the included and excluded participants.

Measures

Resilience. Resilience levels were assessed with the Brief Resilience Scale (BRS) [37] at baseline. Participants rated their personal resilience across 6 items on a 5-point scale (1 = *Strongly disagree* to 5 = *Strongly agree*). Three items were positively worded (e.g., *"I tend to bounce back quickly after hard times*") and three items were negatively worded and reverse scored (e.g., *"I have a hard time making it through stressful events"*). A total score was calculated to reflect overall resilience, with higher scores reflecting higher levels of resilience. The scale showed good internal consistency in the current study at baseline (Cronbach's alpha = .89).

Burnout. Burnout was examined through the Burnout Assessment Tool short version (BAT-12) [38], a 12-item version of the original 23-item questionnaire, which covers four dimensions of burnout: exhaustion (e.g., *"at work, I feel mentally exhausted"*), mental distance (e.g., *"I struggle to find any enthusiasm for my work"),* cognitive impairment (e.g., "*when I'm working, I have trouble concentrating"),* and emotional impairment (e.g., *"at work, I feel unable to control my emotions").*

Each statement was rated on a 5-point scale (1= *never* to 5 = *always*). A mean score was calculated to reflect overall burnout. The scale showed good internal consistency in the current study at all three timepoints (Cronbach's alpha = .91; .89; .92).

Mental well-being. Mental well-being was examined using the Warwick–Edinburgh Mental Wellbeing Scale (WEMWBS) [39]. The 14-item scale included statements covering emotional and functioning aspects of mental well-being. Participants rated how frequently each statement applied to them in the previous two weeks (e.g., "*I've been feeling useful*") on a 5-point scale (1= *none of the time* to 5 = *all of the time*). A total score was obtained by summing all items, with greater scores indicating greater well-being. In the UK, the general population has a mean of 51 with a standard deviation of 7. The scale showed good internal consistency in the current study at all three timepoints (Cronbach's alpha = .93; .94; .95).

Control variables. To control for potential confounding variables, we examined the following control variables which were chosen based on existing literature and expert opinion: age, sex, ethnicity, relationship status, length of time since professional registration, support from colleagues/supervisors/family & friends, COVID infection (yes/no).

Data analysis plan

The analysis plan was pre-registered (https://osf.io/audb8) and data were analysed using Stata 17 [40]. All deviations from this plan are outlined below. To examine the associations between resilience and burnout, as well as mental well-being, we used mixed effect modelling for the two outcome variables separately. To account for the different stages of the pandemic in which the baseline survey was completed, we stratified by time of baseline completion to account for different stages of the pandemic: initial peak (April 2020 – June 2020; n = 543), initial easing of restrictions (July 2020 – September 2020; n = 1,098), second peak (October 2020 – January 2021, n = 1,648). However, as results were largely the same across the three groups, we ran all analyses in the whole sample, controlling for time of baseline completion.

A three-level structure was modelled for each with time-invariant NHS trust at the third level, participant information (i.e., between-subject) at the second level and time-variant repeated measures (i.e., within-subject) at the first level. The analysis first considered the effects of time for each outcome variable. That is, growth modelling of 'Time' (i.e., baseline/6-month follow-up/12-month follow-up) was modelled as a linear, or quadratic trend to determine a pattern of change over-time. For most models a linear trend was significant, except for some of the well-being analyses where neither trend was significant. In these instances, we decided to continue with a linear trend as the most appropriate option.

In the second part of the analysis, we added socio-demographic control variables (e.g., age, sex, education) to the model, one at a time, both as a main effect and interaction with time. The model indices, including AIC, BIC, and $\chi 2$ log-likelihood comparisons, were used to determine whether a particular predictor substantially improves the model and should be retained as a control variable. However, we deviated from our pre-registered analyses and retained some control variables even if they did not improve model fit, for consistency between analyses.

Lastly, the final model was fitted by including all relevant control variables and resilience at baseline to the model to determine whether resilience at baseline predicted change in burnout and mental well-being over time. Resilience was included both as a main effect and interaction with time. Missing data was dealt with using maximum likelihood imputation.

Based on previous analysis from the NHS CHECK study, we used sampling weights to account for sampling bias. The sampling procedure used population-level demographic data provided by each participating Trust's HR department as of April 2020, such as the number of employees and the age, sex, and ethnicity composition of the workforce, and a breakdown by job role. This information was used to calculate a response rate within each Trust. Response weights were generated using a raking algorithm based on age, sex, ethnicity, and role, using the full baseline cohort. Missing data in the weighting process were imputed using the 5th nearest neighbour (kNN) algorithm. Finally,

individual data was weighted by Trust size, and demographic data (ethnicity, categorical age, role and sex).

Ethics

Ethical approval for the NHS CHECK study was granted by the Health Research Authority (reference: 20/HRA/2107, IRAS: 282686) and local Trust Research and Development approval. Participants' written consent was obtained at each study wave.

Results

Sample characteristics

Table 1 outlines the demographic characteristics of the current sample at baseline. The sample was predominantly female and White. The sample was diverse in terms of job roles, comprised mostly of mental health nurses (unweighted: *n* = 1,178; 35.8%), psychologists (unweighted: *n* = 845; 25.7%), healthcare/nursing assistants (unweighted: *n* = 376; 11.4%), doctors /psychiatrists (unweighted: *n* = 295; 9.0%), and occupational therapists (unweighted: *n* = 239; 7.3%).

Table 1. Sample Characteristics at Baseline.

	Group			
	Initial peak Apr-Jun 2020 N (%)	Initial easing Jul-Sep 2020 N (%)	Second peak Oct 2020-Jan 2021 N (%)	Total
Sex				
Female	443 (81.58)	881 (80.24)	1,321 (80.26)	2,645 (80.47)
Male	100 (18.42)	213 (19.40)	316 (19.20)	629 (19.14)
Ethnicity				
White	464 (85.45)	1,007 (91.88)	1,522 (92.80)	2,993 (91.28)
Black	24 (4.42)	22 (2.01)	38 (2.32)	84 (2.56)
Asian	38 (7.00)	34 (3.10)	36 (2.20)	108 (3.29)
Mixed/multiple racial or ethnic groups	15 (2.76)	24 (2.19)	39 (2.38)	78 (2.38)
Age (years)				
≤30	116 (21.76)	216 (20.47)	309 (19.54)	641 (20.23)

31-40	164 (30.77)	250 (23.70)	331 (20.94)	745 (23.51)
41-50	131 (24.58)	298 (28.25)	425 (26.88)	854 (26.95)
51-60	94 (17.64)	238 (22.56)	420 (26.57)	752 (23.73)
≥61	28 (5.25)	53 (5.02)	96 (6.07)	177 (5.59)
Relationship status				
Married/Civil partnership	226 (41.62)	528 (48.22)	764 (46.61)	1,518 (46.32)
Co-habiting/In a relationship	172 (31.68)	350 (31.96)	465 (28.37)	987 (30.12)
Divorced/ separated/ widowed	29 (5.34)	73 (6.67)	156 (9.52)	258 (7.87)
Single	116 (21.36)	144 (13.15)	254 (15.50)	514 (15.69)
Time since professional registration				
< 1 year	23 (4.25)	58 (5.30)	88 (5.35)	169 (5.15)
1 - 2 years	38 (7.02)	70 (6.39)	119 (7.23)	227 (6.92)
3 - 5 years	84 (15.53)	114 (10.41)	167 (10.15)	365 (11.12)
6 - 10 years	90 (16.64)	142 (12.97)	203 (12.34)	435 (13.26)
11 - 20 years	121 (22.37)	231 (21.10)	306 (18.60)	658 (20.05)
21 - 29 years	73 (13.49)	141 (12.88)	214 (13.01)	428 (13.04)
≥30 years	50 (9.24)	136 (12.42)	199 (12.10)	385 (11.73)
Not applicable	62 (11.46)	203 (18.54)	349 (21.22)	614 (18.71)
COVID -19 symptoms				
No	293 (54.77)	660 (60.94)	1,073 (66.07)	2,026 (62.49)
Yes	159 (29.72)	306 (28.25)	427 (26.29)	892 (27.51)
Unsure	83 (15.51)	117 (10.80)	124 (7.64)	324 (9.99)
Resilience (mean score, SD)	21.43 (4.61)	20.65 (4.64)	20.83 (4.79)	20.87 (4.72)
Burnout (mean score, SD)	2.21 (0.63)	2.26 (0.67)	2.21 (0.68)	2.23 (0.67)
Mental well-being (mean score, SD)	45.63 (8.52)	44.63 (8.75)	44.40 (9.27)	44.68 (8.98)

SD = Standard deviation. The following groups were included under each ethnicity category: White (White English/Welsh/Scottish/Northern Irish/British; White Irish; White Gypsy or Irish Traveller; Any other White background); Black (Black African; Black Caribbean; Any other Black background); Asian (Indian; Pakistani, Bangladeshi; Chinese; Any other Asian background); Mixed/multiple racial or ethnic groups (Mixed White and Black Caribbean; Mixed White and Black African; Mixed White and Asian; Any other Mixed/Multiple ethnic background).

Burnout

A significant linear trend of increasing burnout was observed over time ($p < .001$). Resilience at baseline significantly predicted lower levels of burnout (B = -0.06 [-0.06; -0.05], $p < .001$). Additionally, there was a significant interaction with time (B = .01 [.01, .01], $p < .001$), whereby

people with higher resilience levels at baseline had the steepest increases in burnout. Simple slope analyses were conducted to probe the interaction further. That is, we tested the significance of three different slopes: low resilience (one or more standard deviations below the sample mean), average resilience (within one standard deviation of the sample mean), and high resilience (one or more standard deviations above the sample mean). Results are outlined in Fig 1. As can be seen, slopes were steepest in the high resilience group indicating that this group reported the greatest increases in burnout over time.

Fig 1. Burnout scores over a 12-month period with 95% confidence intervals in the overall sample. Findings are adjusted for time of baseline completion, ethnicity, relationship status, time since professional registration, and Covid-19 symptoms at baseline.

Results stratified by group were similar, resilience at baseline significantly predicted lower levels of burnout in the initial peak group (B = -0.07 [-0.07; -0.06], $p < .001$), the initial easing group (B = -0.06 [-0.08; -0.05], $p < .001$), and the second peak group (B = -0.05 [-0.07; -0.04], $p < .001$). A significant interaction with time was observed in the initial peak group (B = .01 [.00, .02], $p = .020$), and second peak group (B = .01 [.00, .01], $p = .007$), but not in the initial easing group (B = .01 [-.00, .02], $p = .154$). Detailed model coefficients are reported in S1 Table.

Mental well-being

No clear trend regarding mental well-being in relation to resilience was observed over time in the overall sample. Within the initial easing group (July-Sept 2020) there was a significant linear trend for decreasing mental well-being (p =.002), however, no significant trend emerged for the initial peak group (p = .489) or second peak group (p =.638). After inspecting the data, we continued with a linear trend model. As a first step for the analyses, we examined significant covariates. Details regarding the covariate selection process are reported in S2 Appendix. After considering both

significant predictors and model fit indices, the final analyses controlled for age, ethnicity, relationship status, time since professional registration, family support, manager support, colleague support, and COVID-19 symptoms.

Resilience at baseline significantly predicted lower levels of mental well-being (B = 0.78 [0.66; 0.90], $p < .001$). Additionally, there was a significant interaction with time (B = -0.08 [0.66; 0.90], $p < .001$), whereby decreases in well-being were present for those with high resilience only. Simple slope analyses were conducted and are reported in Fig 2.

Fig 2. Well-being scores over a 12-month period with 95% confidence intervals in the overall sample. Findings are adjusted for time of baseline completion, ethnicity, relationship status, age, time since professional registration, family support, manager support, colleague support, and Covid-19 symptoms at baseline.

Results stratified by group were somewhat similar, whereby resilience at baseline significantly predicted lower levels of mental well-being in the initial peak group (April-June 2020; B = 0.72 [0.42; 0.24], $p < .001$), the initial easing group (July-Sept 2020; B = 0.77 [0.60; 0.94], $p < .001$), and the second peak group (Oct 2020-Jan 2021; B = 0.79 [0.64; 0.93], $p < .001$). However, there were no significant interactions with time for any of the three groups and hence no further analyses (i.e., simple slope analyses) were conducted. Detailed model coefficients are reported in S2 Appendix.

Discussion

This study examined the long-term changes in burnout and mental well-being among NHS mental health staff throughout the COVID-19 pandemic, and their relationship with resilience. Four key findings were observed. First, there was an increase in burnout over time among all clinical mental health staff. Second, higher initial resilience at baseline predicted lower burnout at 6 and 12 months. Third, staff with higher initial resilience levels showed the steepest increases in burnout

over time. Despite this, they remained the least at risk of burnout at 12-month follow-up. Fourth, lower resilience levels at baseline predicted lower mental well-being over 12 months with those reporting higher resilience at the start of the pandemic reporting a decrease in well-being over time.

Our findings show that burnout levels continued to increase among mental health staff throughout the pandemic, which is in line with the increasing workload pressures, scarcer resources, and the prolonged effects of the COVID-19 pandemic on issues such as long waiting lists, staff absences, and the anticipation of an increased need for mental health care [41, 8]. Additionally, there are concerns that pre-existing issues relating to healthcare staff's working conditions, including inadequate resourcing, equipment, and remuneration, have been exacerbated and may have also shaped workers' responses to the pressures of the pandemic [42]. Although the lack of existing literature on the prospective associations between resilience and burnout and mental well-being among mental health staff poses a challenge to direct comparisons with our findings, comparisons can be drawn with cross-sectional research. Despite staff with higher resilience levels showing lower burnout overall, these findings do not support some previous research suggesting that healthcare workers may have 'bounced back' from the initial peak of the pandemic [24, 27], although this may be because of the ongoing challenging work environment effectively meaning that staff cannot bounce back to a more usual, less stressful, workplace.

We also found that staff with higher resilience levels showed the most prominent increases in burnout, indicating that the most highly resilient staff experienced the most negative effects of burnout during the pandemic. Staff with initially high resilience levels were also the only ones to report decreases in mental well-being, whereas staff with low or average resilience reported no change in well-being over time. Whilst this appears contrary to the definitions of resilience, which describes an adaptation to adverse experiences and thus we should expect that lower rates of burnout over time would have been observed among the most resilient staff [16], there may be several explanations for this. Firstly, as we state above, even the most resilient staff may have

difficulties bouncing back in a work environment that is constantly changing due to evolving work-related stressors. For instance, after dealing with the initial transition to COVID-19 care, the NHS had to deal with staff shortages, as well as having to catch up with missed care provision. Secondly, whilst mental health care staff who reported initially higher levels of resilience experienced the greatest deterioration in burnout and well-being scores, this may have been because their initial low score provided scope for large changes. We note that despite experiencing the greatest deterioration in burnout and well-being, they remained significantly less burnt out and with better well-being compared to staff with initially lower levels of resilience.

Therefore, although more resilient staff were doing better overall, our findings suggest that other factors could be at play regarding mental health staff burnout during the pandemic and may remain present even beyond the acute phase of COVID-19. Indeed, a recent overview of systematic reviews found strong quality evidence of organisational-level interventions targeting factors such as leadership development and improving work-life balance, either standalone or combined with individual ones, reducing burnout in healthcare workers [43]. As such, resilience only explains part of the picture when it comes to mental health staff burnout and well-being during peak times of stress. Taking together the current low-certainty evidence for the efficacy of resilience interventions for healthcare staff, mixed cross-sectional findings, and our longitudinal study findings, resilience as a standalone target factor is likely not enough to prevent or support those experiencing burnout. Instead, structural factors such as understaffing, access to adequate time off and staff meals, play a more important role in staff burnout and well-being and have been emphasised by healthcare workers themselves [42].

Thus, while resilience interventions may be useful for staff with lower resilience levels, there are concerns that focusing on resilience, particularly among those who are already highly resilient, may be counter-productive, and overshadow organisational factors involved in staff well-being. These groups of staff may feel personally responsible for their struggle to cope, having

already developed resilience skills yet still feeling increasingly burnt out. Our findings indicate that resilience-focused interventions may have some benefit on improving staff well-being and limited benefits on decreasing burnout; instead, other avenues should be explored to tackle burnout among this staff group. In the meantime, it is crucial that mental health staff, particularly those who have already developed high resilience, have adequate resources and managerial support available. Further research should investigate different burnout trajectories and potential factors which may affect groups of mental health staff differently.

While the current study has important practical implications for the overall well-being of mental health staff, some limitations should be noted. Since the data in the current study covered the period up until the second peak of the COVID-19 pandemic, data collected at later points are needed to clarify the impact of resilience on burnout and well-being over time as the pandemic progressed. Furthermore, exploring whether a change in resilience subsequently affects a change in burnout and well-being would be a helpful step towards understanding the importance of resilience interventions, considering the evolving workplace factors affecting mental health staff following the COVID-19 pandemic.

In conclusion, the current study showed a steady increase in burnout and slight decline in well-being among mental health staff throughout the pandemic. Staff with high levels of resilience tended to report the lowest levels of burnout and highest levels of mental well-being overall, however, they also reported the steepest increases in burnout. This indicates that more support is needed to prevent burnout, especially in highly resilient mental health staff who are unlikely to benefit from existing resilience interventions.

Acknowledgements: We wish to acknowledge the National Institute of Health and Care Research (NIHR) Applied Research Collaboration (ARC) National NHS and Social Care Workforce Group, with the following ARCs: North Thames, East Midlands, East of England, South West Peninsula, South London, West, North West Coast, Yorkshire and Humber, and North East and North Cumbria. They enabled the set-up of the national network of participating hospital sites and aided the research team to recruit effectively during the COVID-19 pandemic.

This work is independent research supported by the National Institute for Health Research (NIHR) Applied Research Collaboration (ARC) North Thames and the National Institute for Health and Care Research Health Protection Research Unit (NIHR HPRU) in Emergency Preparedness and Response, a partnership between the UK Health Security Agency, King's College London and the University of East Anglia. The views expressed are those of the author(s) and not necessarily those of the NIHR, UKHSA or the Department of Health and Social Care. For the purpose of open access, the author has applied [a Creative Commons Attribution (CC BY) license] [an 'Open Government License'] to any Author Accepted Manuscript version arising.

The NHS CHECK consortium includes the following site leads: Siobhan Coleman, Sean Cross, Amy Dewar, Chris Dickens, Frances Farnworth, Adam Gordon, Charles Goss, Jessica Harvey, Nusrat Husain, Peter Jones, Damien Longson, Paul Moran, Jesus Perez, Mark Pietroni, Ian Smith, Tayyeb Tahir, Peter Trigwell, Jeremy Turner, Julian Walker, Scott Weich, Ashley Wilkie.

The NHS CHECK consortium includes the following co-investigators and collaborators: Peter Aitken, Ewan Carr, Anthony David, Mary Jane Doherty, Sarah Dorrington, Rosie Duncan, Sam Gnanapragasam, Cerisse Gunasinghe, Stephani Hatch, Danielle Lamb, Daniel Leightley, Ira Madan, Richard Morriss, Isabel McMullen, Dominic Murphy, Martin Parsons, Catherine Polling, Alexandra Pollitt, Anne-Marie Rafferty, Rebecca Rhead, Danai Serfioti, Chloe Simela, Charlotte Wilson Jones.

References:

1. Haldane V, de Foo C, Abdalla SM, Jung AS, Tan M, Wu S, et al. Health systems resilience in managing the COVID-19 pandemic: lessons from 28 countries. Nat Med. 2021;27(6):964–80. https://doi.org/10.1038/s41591-021-01381-y

2. Moreno C, Wykes T, Galderisi S, Nordentoft M, Crossley N, Jones N, et al. How mental health care should change as a consequence of the COVID-19 pandemic. Lancet Psychiatry. 2020;7(9):813–24. https://doi.org/10.1016/S2215-0366(20)30307-2

3. Duden GS, Gersdorf S, Trautmann K, Steinhart I, Riedel-Heller S, Stengler K. LeiP#netz 2.0: mapping COVID-19-related changes in mental health services in the German city of Leipzig. Soc Psychiatry Psychiatr Epidemiol. 2022;57:1531–41. https://doi.org/10.1007/s00127-022-02274-2

4. Li L. Challenges and priorities in responding to covid-19 in inpatient psychiatry. Psychiatr Serv. 2020;71(6):624-6. https://doi.org/10.1176/appi.ps.202000166

5. Percudani M, Corradin M, Moreno M, Indelicato A, Vita A. Mental Health Services in Lombardy during COVID-19 outbreak. Psychiatry Res. 2020;288:112980. https://doi.org/10.1016/j.psychres.2020.112980

6. Starace F, Ferrara M. COVID-19 disease Emergency Operational Instructions for Mental Health Departments issued by the Italian Society of Epidemiological Psychiatry. Epidemiol Psychiatr Sci. 2020. https://doi.org/10.1017/S2045796020000372

7. Mezzina R, Sashidharan SP, Rosen A, Killaspy H, Saraceno B. Mental health at the age of coronavirus: time for change. Soc Psychiatry Psychiatr Epidemiol. 2020;55(8):965–8. https://doi.org/10.1007/s00127-020-01886-w

8. Sammons MT, Elchert DM, Martin JN. Mental Health Service Provision During COVID-19: Results of the Third Survey of Licensed Psychologists. J Health Serv Psychol. 2021;47(3):119–27. https://doi.org/10.1007/s42843-021-00044-3

9. Luthar SS. Resilience in development: a synthesis of research across five decades. In: Cicchetti D, Cohen DJ, editors. Developmental psychopathology: risk, disorder, and adaptation. Wiley; 2006. p. 740-95.

10. Yang Y, Hayes JA. Causes and consequences of burnout among mental health professionals: A practice-oriented review of recent empirical literature. Psychotherapy. 2020;57(3):426–36. https://doi.org/10.1037/pst0000317

11. World Health Organization (WHO). International Classification of Diseases, Eleventh Revision (ICD-11). 2019.

12. Joshi G, Sharma G. Burnout: A risk factor amongst mental health professionals during COVID-19. Asian J Psychiatr. 2020;54. https://doi.org/10.1016/j.ajp.2020.102300

13. Foye U, Dalton-Locke C, Harju-Seppänen J, Lane R, Beames L, Vera San Juan N, et al. How has COVID-19 affected mental health nurses and the delivery of mental health nursing care in the UK? Results of a mixed-methods study. J Psychiatr Ment Health Nurs. 2021;28(2):126–37. https://doi.org/10.1111/jpm.12745

14. Rapisarda F, Bergeron N, Dufour MM, Guay S, Geoffrion S. Longitudinal assessment and determinants of short-term and longer-term psychological distress in a sample of healthcare workers during the COVID-19 pandemic in Quebec, Canada. Front Psychiatry. 2023;14. https://doi.org/10.3389/fpsyt.2023.1112184

15. Brooks S, Amlôt R, Rubin GJ, Greenberg N. Psychological resilience and post-traumatic growth in disaster-exposed organisations: Overview of the literature. J R Army Med Corps. 2018. https://doi.org/10.1136/jramc-2017-000876

16. Luthar SS, Cicchetti D. The construct of resilience: Implications for interventions and social policies. Dev Psychopathol. 2000;12(4):857–85. https://doi.org/10.1017/S0954579400004156

17. Chan ACY, Piehler TF, Ho GWK. Resilience and mental health during the COVID-19 pandemic: Findings from Minnesota and Hong Kong. J Affect Disord. 2021;295:771–80. https://doi.org/10.1016/j.jad.2021.08.144

18. De los Santos JAA, Labrague LJ. The impact of fear of COVID-19 on job stress, and turnover intentions of frontline nurses in the community: A cross-sectional study in the Philippines. Traumatology. 2021;27(1):52–9. https://doi.org/10.1037/trm0000294

19. Setiawati Y, Wahyuhadi J, Joestandari F, Maramis MM, Atika A. Anxiety and resilience of healthcare workers during COVID-19 pandemic in Indonesia. J Multidiscip Healthc. 2021;14:1–8. https://doi.org/10.2147/JMDH.S276655

20. Barzilay R, Moore TM, Greenberg DM, DiDomenico GE, Brown LA, White LK, et al. Resilience, COVID-19-related stress, anxiety and depression during the pandemic in a large population enriched for healthcare providers. Transl Psychiatry. 2020;10(1). https://doi.org/10.1038/s41398-020-00982-4

21. Pigati PA da S, Righetti RF, Dourado VZ, Nisiaymamoto BTC, Saraiva-Romanholo BM, Tibério I de FL C. Resilience Improves the Quality of Life and Subjective Happiness of Physiotherapists during the COVID-19 Pandemic. Int J Environ Res Public Health. 2022;19(14):8720. https://doi.org/10.3390/ijerph19148720

22. Fletcher D, Sarkar M. Psychological resilience: A review and critique of definitions, concepts, and theory. Eur Psychol. 2013;18(1):12–23. https://doi.org/10.1027/1016-9040/a000124

23. Hines SE, Chin KH, Glick DR, Wickwire EM. Trends in moral injury, distress, and resilience factors among healthcare workers at the beginning of the covid-19 pandemic. Int J Environ Res Public Health. 2021;18(2):1–11. https://doi.org/10.3390/ijerph18020488

24. García-Fernández L, Romero-Ferreiro V, Rodríguez V, Alvarez-Mon MA, Lahera G, Rodriguez-Jimenez R. What about mental health after one year of COVID-19 pandemic? A comparison with the initial peak. J Psychiatr Res. 2022;153:104–8. https://doi.org/10.1016/j.jpsychires.2022.07.010

25. Gilleen J, Santaolalla A, Valdearenas L, Salice C, Fusté M. Impact of the COVID-19 pandemic on the mental health and well-being of UK healthcare workers. BJPsych Open. 2021;7(3). https://doi.org/10.1192/bjo.2021.42

26. Labrague LJ. Psychological resilience, coping behaviours and social support among health care workers during the COVID-19 pandemic: A systematic review of quantitative studies. J Nurs Manag. 2021;29(7):1893–905. https://doi.org/10.1111/jonm.13336

27. Riehm KE, Holingue C, Smail EJ, Kapteyn A, Bennett D, Thrul J, et al. Trajectories of Mental Distress Among U.S. Adults During the COVID-19 Pandemic. Ann Behav Med. 2021;55(2):93–102. https://doi.org/10.1093/abm/kaaa126

28. Albott CS, Wozniak JR, McGlinch BP, Wall MH, Gold BS, Vinogradov S. Battle Buddies: Rapid Deployment of a Psychological Resilience Intervention for Health Care Workers During the

COVID-19 Pandemic. Anesth Analg. 2020;131(1):43–54. https://doi.org/10.1213/ANE.0000000000004912

29. Rosen B, Preisman M, Read H, Chaukos D, Greenberg RA, Jeffs L, et al. Providers' perspectives on implementing resilience coaching for healthcare workers during the COVID-19 pandemic. BMC Health Serv Res. 2022;22(1):780. https://doi.org/10.1186/s12913-022-08114-4

30. DeTore NR, Sylvia L, Park ER, Burke A, Levison JH, Shannon A, et al. Promoting resilience in healthcare workers during the COVID-19 pandemic with a brief online intervention. J Psychiatr Res. 2022;146:228-233. https://doi.org/10.1016/j.jpsychires.2021.11.011

31. Pollock A, Campbell P, Cheyne J, Cowie J, Davis B, McCallum J, et al. Interventions to support the resilience and mental health of frontline health and social care professionals during and after a disease outbreak, epidemic or pandemic: a mixed methods systematic review. Cochrane Database Syst Rev. 2020;(11). https://doi.org/10.1002/14651858.CD013779

32. Traynor M. Guest editorial: What's wrong with resilience. J Res Nurs. 2018;23(1):5-8. https://doi.org/10.1177/1744987117751458

33. Rosen B, Preisman M, Read H, Chaukos D, Greenberg RA, Jeffs L, et al. Resilience coaching for healthcare workers: Experiences of receiving collegial support during the COVID-19 pandemic. Gen Hosp Psychiatry. 2022;75:83-87. https://doi.org/10.1016/j.genhosppsych.2022.02.003

34. Billings J, Ching BCF, Gkofa V, Greene T, Bloomfield M. Experiences of frontline healthcare workers and their views about support during COVID-19 and previous pandemics: a systematic review and qualitative meta-synthesis. BMC Health Serv Res. 2021;21(1):923. https://doi.org/10.1186/s12913-021-06917-z

35. Litz BT, Stein N, Delaney E, Lebowitz L, Nash WP, Silva C, et al. Moral injury and moral repair in war veterans: A preliminary model and intervention strategy. Clin Psychol Rev. 2009;29(8):695-706. https://doi.org/10.1016/j.cpr.2009.07.003

36. Lamb D, Greenberg N, Hotopf M, et al. NHS CHECK: protocol for a cohort study investigating the psychosocial impact of the COVID-19 pandemic on healthcare workers. BMJ Open. 2021;11. https://doi.org/10.1136/bmjopen-2021-051687

37. Smith BW, Dalen J, Wiggins K, Tooley E, Christopher P, Bernard J. The Brief Resilience Scale: Assessing the Ability to Bounce Back. Int J Behav Med. 2008;15:194-200. https://doi.org/10.1080/10705500802222972

38. Hadžibajramović E, Schaufeli W, de Witte H. Shortening of the Burnout Assessment Tool (BAT)—from 23 to 12 items using content and Rasch analysis. BMC Public Health. 2022;22(1):1-16. https://doi.org/10.1186/s12889-022-12946-y

39. Tennant R, Hiller L, Fishwick R, Platt S, Joseph S, Weich S, et al. The Warwick-Edinburgh mental well-being scale (WEMWBS): Development and UK validation. Health Qual Life Outcomes. 2007;5(1):1-13. https://doi.org/10.1186/1477-7525-5-63

40. StataCorp. Stata Statistical Software: Release 17. College Station, TX: StataCorp LLC; 2021.

41. O'Connor RC, Wetherall K, Cleare S, McClelland H, Melson AJ, Niedzwiedz CL, et al. Mental health and well-being during the COVID-19 pandemic: longitudinal analyses of adults in the UK COVID-19 Mental Health & Wellbeing study. Br J Psychiatry. 2021;218(6):326-333. https://doi.org/10.1192/bjp.2020.212

42. Vera San Juan N, Aceituno D, Djellouli N, Sumray K, Regenold N, Syversen A, et al. Mental health and well-being of healthcare workers during the COVID-19 pandemic in the UK: contrasting guidelines with experiences in practice. BJPsych Open. 2021;7(1). https://doi.org/10.1192/bjo.2020.148

43. Aust B, Møller JL, Nordentoft M, Frydendall KB, Bengtsen E, Jensen AB, et al. How effective are organizational-level interventions in improving the psychosocial work environment, health, and retention of workers? A systematic overview of systematic reviews. Scand J Work Environ Health. 2023;49(5):315-329. https://doi.org/10.5271/sjweh.4097

Figure 1

Click here to access/download;Figure;Fig 1.tif

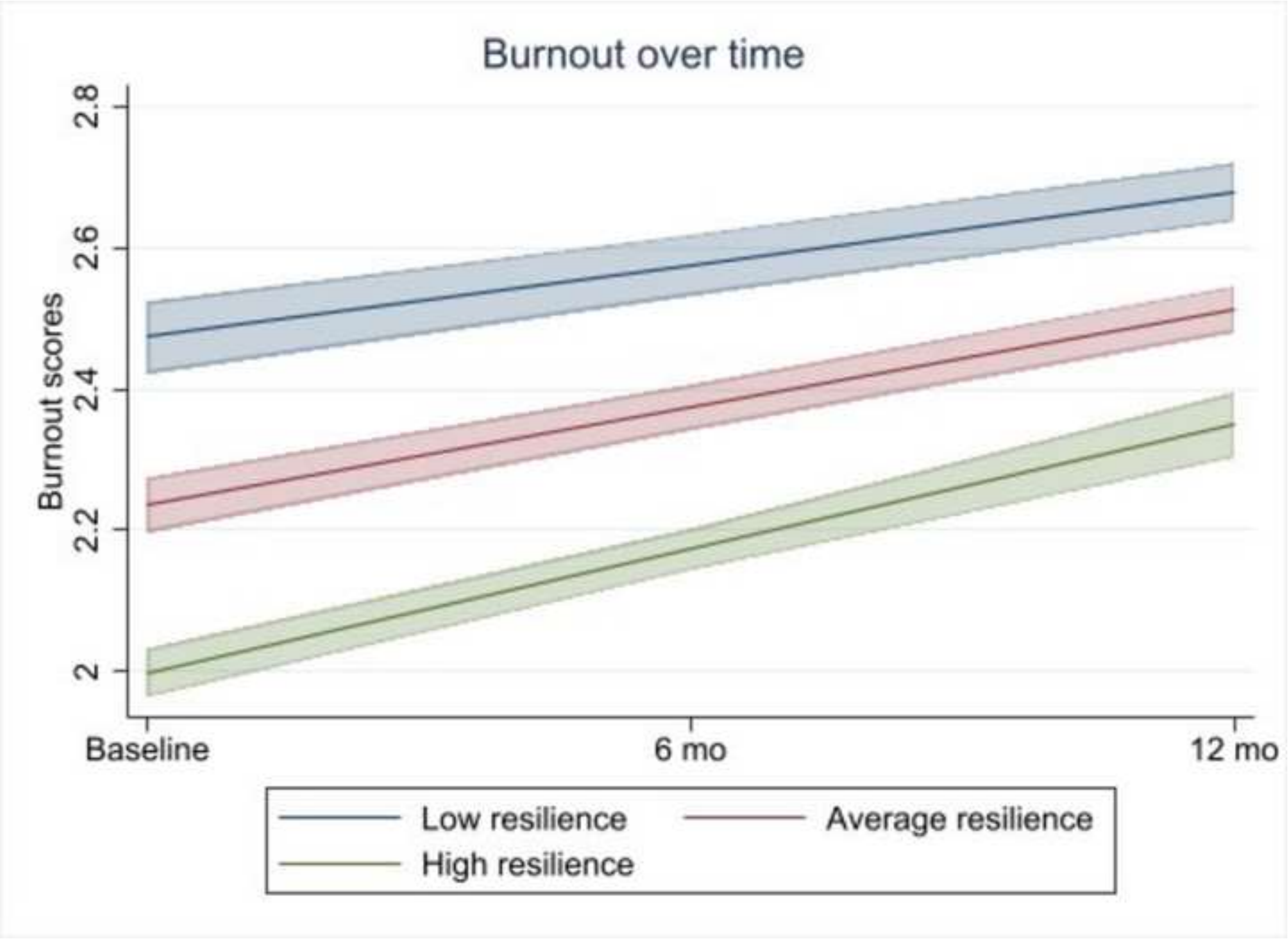

Figure 2

Click here to access/download;Figure;Fig 2.tif

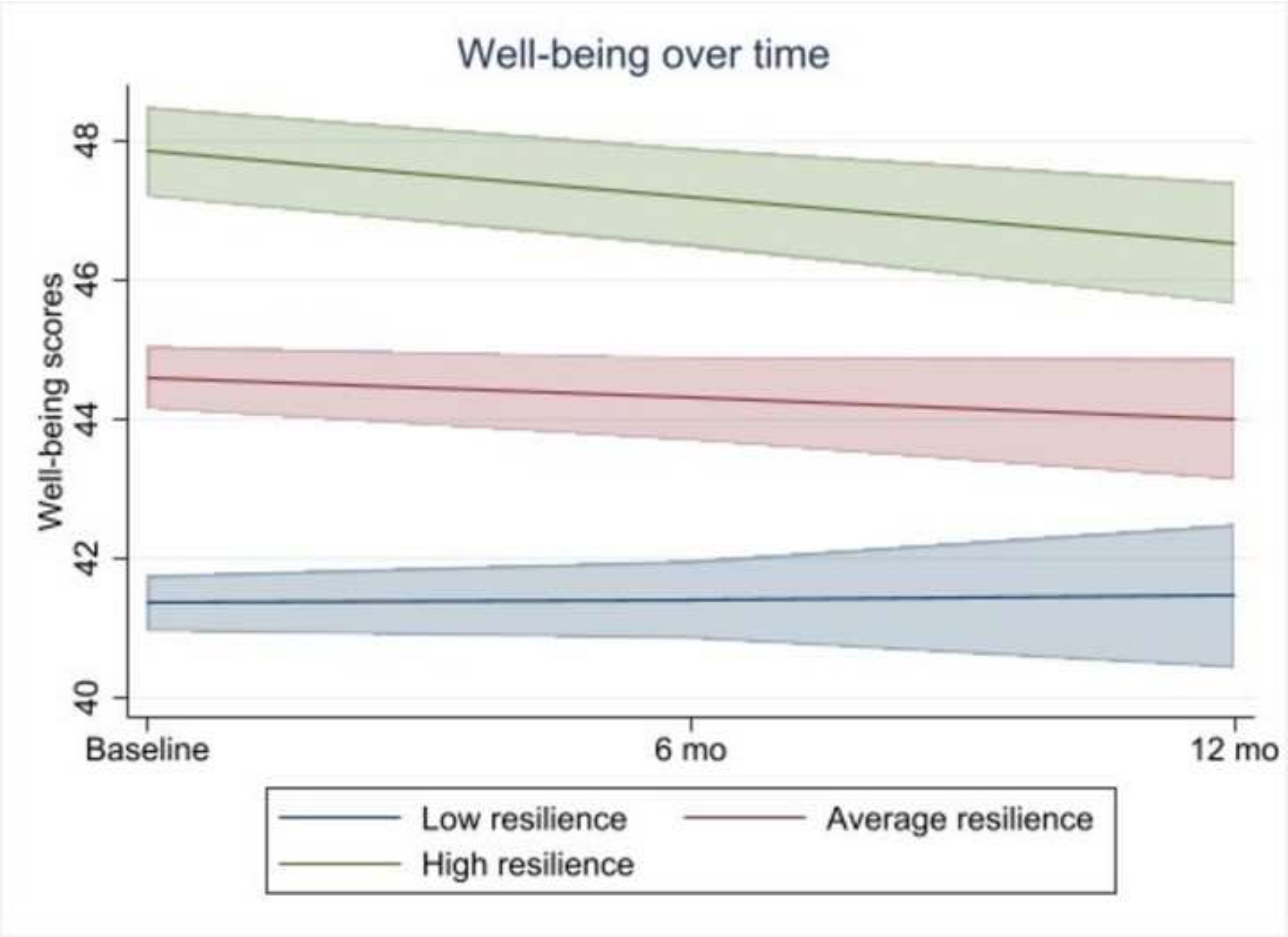

Supplementary Table

Click here to access/download
Supporting Information
S1 Table.docx

Click here to access/download
Supporting Information
S2 Appendix.docx

Manuscript PONE-D-24-24436

Response to Reviewers

Dear Editor in Chief,

We would like to re-submit this manuscript in order to address an oversight in our previous submission. We appreciate the time taken to provide feedback on our manuscript. Please see below our response to the concerns raised.

Reviewer's concern:

"We recognize that you obtained ethical approval from your local ethics committee after you began your study: your ethics approval document is dated 24 April 2020, while you began recruiting participants on 01 April 2020. You also state that informed consent was obtained from all participants, which indicates that the data were prospectively collected for research purposes. For human subjects research, PLOS ONE requires that authors obtain ethics committee approval before beginning the study, and we do not accept retrospective ethical approval."

Reponse:

We apologise for this oversight; the dates provided were standardised to the first of each month that data collection had begun which resulted in an error. The corresponding author has now confirmed the correct dates with the project manager and has updated these under 'Participants and procedure' in the Methods section of the re-submitted manuscript. Therefore, ethical approval was indeed obtained before the start of data collection (24/04/2020).

These are:

Wave 1 (Baseline): Between 27/04/2020 and 15/01/2021

Wave 2 (6-month follow-up): Between 27/10/2020 until 15/08/2021

Wave 3 (12-month follow-up): Between 27/04/2021 until 21/02/2022
