# Supplementary material for: Resilience during the COVID-19 pandemic: Associations with changes in burnout and mental well-being among NHS mental health staff in England
Source: PLoS One. 2025 Jul 8;20(7):e0326753. doi: 10.1371/journal.pone.0326753 (PMC12237035; doi:10.1371/journal.pone.0326753)
Supplement: Attachment — Submitted filename: Reponse to Reviewers Letter PONE-D-24-24436.docx [file pone.0326753.s006.docx]

**Manuscript PONE-D-24-24436**

**Response to Reviewers**

Dear Editor in Chief,

We would like to re-submit this manuscript in order to address an oversight in our previous submission. We appreciate the time taken to provide feedback on our manuscript. Please see below our response to the concerns raised.

**Reviewer’s concern:**

“We recognize that you obtained ethical approval from your local ethics committee after you began your study: your ethics approval document is dated 24 April 2020, while you began recruiting participants on 01 April 2020. You also state that informed consent was obtained from all participants, which indicates that the data were prospectively collected for research purposes. For human subjects research, PLOS ONE requires that authors obtain ethics committee approval before beginning the study, and we do not accept retrospective ethical approval.”

**Reponse:**

We apologise for this oversight; the dates provided were standardised to the first of each month that data collection had begun which resulted in an error. The corresponding author has now confirmed the correct dates with the project manager and has updated these under ‘Participants and procedure’ in the Methods section of the re-submitted manuscript. Therefore, ethical approval was indeed obtained before the start of data collection (24/04/2020).

These are:

Wave 1 (Baseline): Between 27/04/2020 and 15/01/2021

Wave 2 (6-month follow-up): Between 27/10/2020 until 15/08/2021

Wave 3 (12-month follow-up): Between 27/04/2021 until 21/02/2022
